# Effects of Different RH Degasser Nozzle Layouts on the Circulating Flow Rate

**DOI:** 10.3390/ma15238476

**Published:** 2022-11-28

**Authors:** Zhenming Zhang, Peng Ma, Jianhong Dong, Min Liu, Yonggang Liu, Chaobin Lai

**Affiliations:** 1 Faculty of Materials Metallurgy and Chemistry, Jiangxi University of Science and Technology, Ganzhou 341000, China; 2 Xinyu Iron & Steel Group Co., Ltd., Xinyu 338001, China; 3 School of Materials Science and Engineering, Hanshan Normal University, Chaozhou 521041, China

**Keywords:** RH refining, nozzle layout, circulating flow rate, water model, mathematical model

## Abstract

The effects of gas flow rate and 22 kinds of nozzle layouts on the circulation flow rate were researched during the RH refining process using a water model and a mathematical model. Numerical simulations agreed with the water model experiment well. The results showed that the circulating flow rate increased with an increase of the gas flow rate. The critical value of the gas flow rate was 2.4 m^3^/h. Out of the 22 kinds of layouts, the 127-87 symmetrical layout was the optimal layout, for which the circulating flow rate reached 29.8 m^3^/h, the area of blind zone was the smallest and the mixing effect of the molten steel was the best. The working stroke and carrying capacity of the bubbles were important factors that affected the circulating flow rate. Among the four types of layouts, when the nozzles were in the one-side layout and the one-row layout, the main factor for improving the circulating flow rate was the working stroke of the bubbles. When nozzles were in the staggered layout and the symmetrical layout, the carrying capacity of the bubbles was the main factor for improving the circulating flow rate. For the same conditions, the carrying capacity of the bubbles had a greater effect on improving the circulating flow rate than the bubble stroke.

## 1. Introduction

The Ruhrstahl-Heraeus (RH) process is one of the most widely used vacuum devices in secondary refining, which plays an important role in deep decarburization, deoxidation, desulfurization, hydrogen and nitrogen removal, alloying, molten steel mixing and inclusion removal. Therefore, the RH process is very complex, involving complicated reactions and gas-liquid two-phase flow in a metallurgical process. Its related research is very widely known. 

Metallurgical processes in the RH degasser are hardly observed and tested due to its limitations of high temperature, being a multiphase system and vacuum conditions. In general, numerical simulations and water modeling were used to study and visualize this fundamental phenomenon in the RH degasser [1,2,3,4]. In constructing a water model, dynamic similarity must be satisfied first [5,6]. It required that the modified Froude number in the model should be equivalent to that in the prototype. Currently, modeling methods by numerical simulation mainly involve a quasi-single-phase model [1,3], the Euler-Lagrange method [7] and the Euler-Euler method [8,9,10,11,12]. In addition, the discrete phase model (DPM) and the fractional volume model (VOF) were combined to simulate the multiphase flow in the RH degasser [9,13,14].

In the RH process, the circulation flow rate of liquid steel plays an extremely important role in determining the productivity of the equipment, because it affects the refining efficiency. Most studies [8,10,15,16,17,18,19] aimed to understand the effects of various operating parameters in the RH process on the circulation flow, including vacuum pressure, gas flow rate, RH vessel and vent size. Jiang F et al. [16] investigated the effects of multi-hole orifices in the upleg snorkel on the bubble behavior during Ruhrstahl–Heraeus refining. Ling et al. [20] reported that as the number of nozzles increased, a value reached an optimum maximum circulation flow rate and minimum mixing time. Dai W et al. [21] compared this with the unexpanded bubble, and found that the expanded bubble contributed a higher circulation rate in SSRF and induced a greater superficial area of free surface in the vacuum chamber, owing to its larger, rising velocity. The circulation rate increased with a higher gas flow rate [1,2,3,7,22,23], lower vacuum pressure [1], larger immersion depth [1,7,19] and larger snorkel diameter [1,16,24,25]. Han et al. [26] reported a maximum circulation flow rate for an injection angle of 12 degrees. 

However, there are few studies on the influence of an argon blowing nozzle layout on the circulating flow rate. In this study, the variation of the circulation flow rate was measured with a water model under different gas flow rates and nozzle layouts. Also, a mathematical model was developed to research the effect of lifting the gas flow rate and the nozzle layout on the circulation flow rate and flow field in the ladle.

## 2. Materials and Methods

### 2.1. RH Water Model

**Figure 1:**
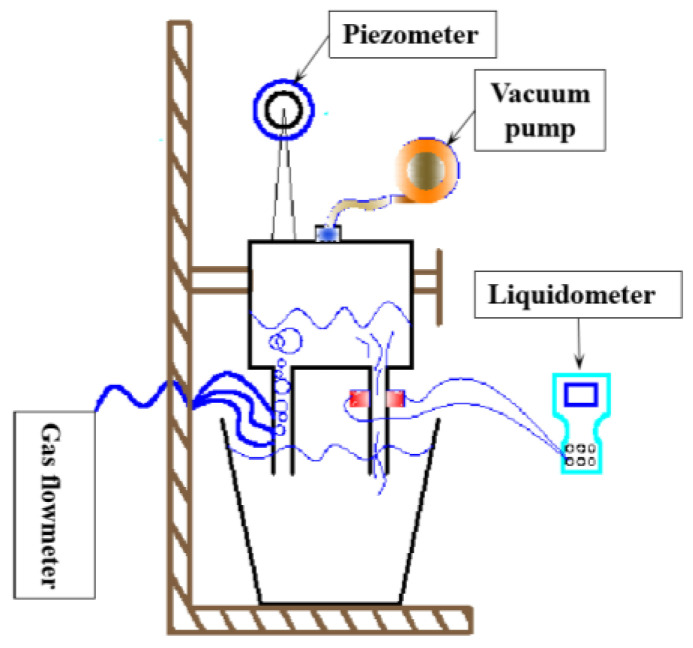
RH water model device.

A water model was designed based on an industrial 120-ton RH degasser with a similarity ratio of 1:3, as shown in Figure 1.

It consisted of a vacuum chamber, ladle water model, air supply system and metering system. According to the time difference method, the circulating flow rate was measured by ultrasonic flowmeter. Bubble buoyancy was the main driving force for the flow in the system. Therefore, the modified Froude constant was selected as the similarity criterion. The model and prototype parameters were converted as follows:

(1) $$Q_{m}=0.0285Q_{P}$$

*Q_m_* and *Q_P_* are the model gas flow rate of the model and the prototype, where the unit is m^3^/h. The main parameters of the model and the prototype are shown in Table 1 and Table 2.

As shown in Table 3, the effects of the gas flow rate and the different nozzle layouts on the circulating flow rate were studied.

The nozzle layouts were divided into 4 categories, namely staggered layout, one-side layout, symmetrical layout and one-row layout. Among them, staggered layout, one-sided layout and symmetrical layout had 6 kinds of layouts, whereas the one-row had 4 kinds of layouts; a total of 22 layout modes are shown in the Table 4.

**Figure 2:**
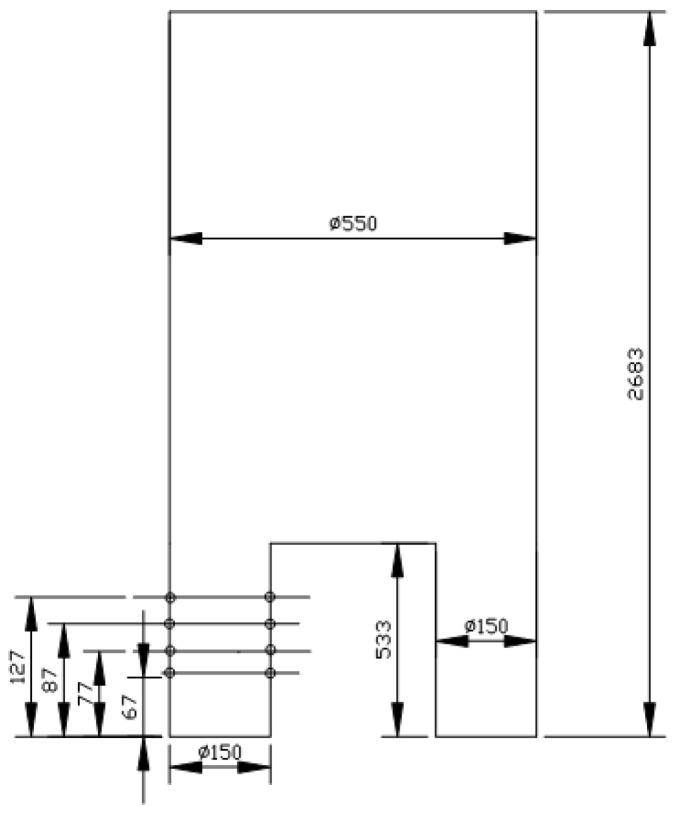
Schematic diagram of the RH vacuum chamber.

There were 16 nozzles in each nozzle arrangement. The numbers 127, 87, 77 and 67 represent the distance between the nozzle and the vacuum chamber, with units mm, as shown in Figure 2.

**Figure 3:**
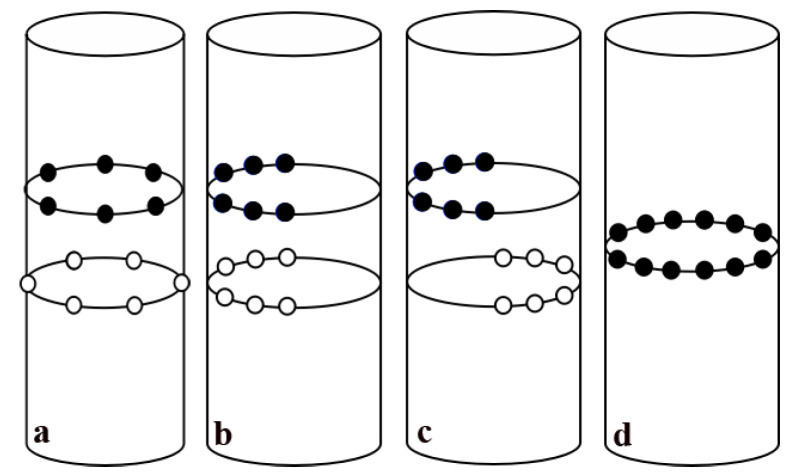
Schematic diagram of the blowhole layout: (**a**) Staggered Layout (type A), (**b**) One-side Layout (type B), (**c**) Symmetrical Layout (type C), (**d**) One-row Layout (type D).

In the staggered layout (type A), nozzles were divided into upper and lower layers and stagger from each other. In the one-side layout (type B), nozzles were distributed on one side of the upleg. In the symmetrical (type C) layout, nozzles were symmetrically arranged in the upper and lower semicircles. In the one-row layout (type D), nozzles were evenly distributed in a circle. The schematic diagram of each nozzle layout is shown in Figure 3. 

### 2.2. Numerical Simulation 

#### 2.2.1. Basic Assumptions 

A mathematical model was established for the flow behavior of molten steel in the RH refining process. To simplify the actual problem, the following basic assumptions were made:

(1) Assuming that the simulated flow process was carried out under isothermal conditions, the temperature of the water and the air was 298 K, regardless of the temperature transfer and the influence of temperature on the phase parameters;
(2) Liquid phase was an incompressible viscous fluid, with no slip at the wall;
(3) The bubble was set as a discrete phase fluid, with a particle size of 5 mm, a rigid sphere and ignoring the rupture and polymerization between bubbles;
(4) The pressure of two phases in the same computational domain was the same.

#### 2.2.2. Model Construction 

**Figure 4:**
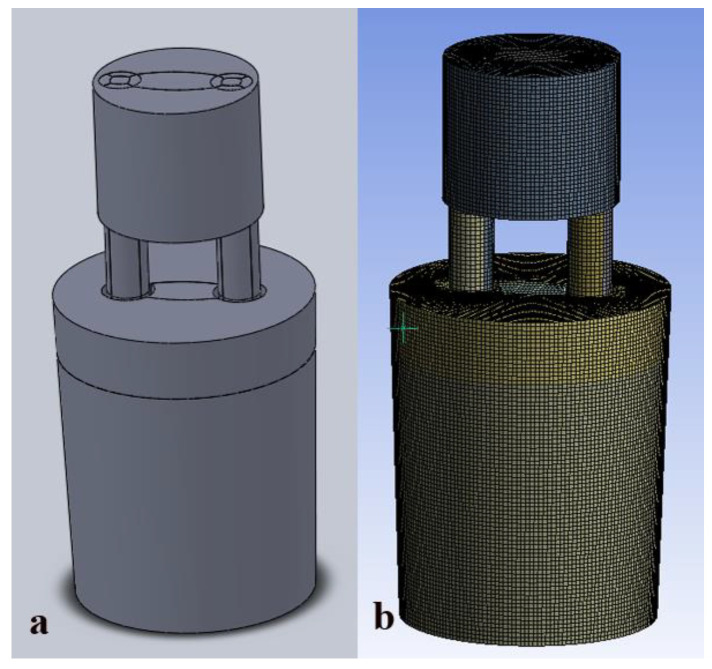
RH refining furnace geometric model and meshing: (**a**) geometric model, (**b**) meshing.

In this paper, SolidWorks software was used to create geometric models. The geometric models were transformed into interface files (Parasolid format) that can be identified by ANSYS Workbench. The whole model was divided into three parts, including the upper cylindrical vacuum chamber, the lower inverted truncated cone ladle and the upleg and downleg. The geometric model is shown in Figure 4.

#### 2.2.3. Control Equation 

In this paper, the Euler-Euler model was used to study the influence of different nozzle layouts on the flow field structure and blind zone of the fluid in the ladle of the RH system.

(1) Volume Fraction

In order to describe the interpenetrated and continuous multiphase flow, the concept of phase volume fraction was introduced, which is represented by *α*. Volume fraction represented the space occupied by each phase, and each phase had its own corresponding momentum conservation equation. 

The volume of *q* phase *V_q_* is defined as:

(2) $$V_{q}=\int_{v}\alpha_{q}dV,$$

Because there were only liquid and gas, the following formula holds for α*_q_* in (2):

(3) $$\;\alpha_{l}+\alpha_{g}=1,$$

The effective density of phase *q* is:

(4) $$V{\overset{⌢}{\rho }}_{q}=\alpha_{q}\cdot \rho_{q},$$

(2) Continuity Equation

In this paper, the liquid phase was set as the main phase and the gas was set as the second phase. The continuity equation of *q* phase is as follows:

(5) $$\frac{\partial }{\partial t}\left(\alpha_{q}\rho_{q}\right)+\nabla \cdot \left(\alpha_{q}\rho_{q}v_{q}\right)=0,$$

where *v_q_* is the velocity of *q* phase; *α_q_* represents the volume fraction of *q* phase; *ρ_q_* denotes the density of *q* phase,

(3) Momentum Conservation Equation

For *q* phase, the momentum conservation equation was as follow:

(6) $$\frac{\partial }{\partial t}\left(\alpha_{q}\rho_{q}\overset{r}{v_{q}}\right)+\nabla \cdot \left(\alpha_{q}\rho_{q}\overset{r}{v_{q}}\overset{r}{v_{q}}\right)=-\alpha_{q}\nabla P+\nabla \cdot \left(\alpha_{q}\mu_{q,eff}\left(\nabla \overset{r}{v_{q}}+\nabla {\overset{r}{v_{q}}}^{T}\right)\right)+\alpha_{q}\rho_{q}\overset{r}{g}+F_{q},$$

In the Equation (6), *P* is the pressure shared by the two phases; $\overset{r}{g},\overset{r}{v_{q}}$ are the gravity acceleration vector and the *q*-phase velocity vector, respectively; *F_q_* is the inter-phase force of *q* phase, which in this paper, mainly refers to the drag force; *μ_q,eff_* is the viscosity coefficient.

#### 2.2.4. Initial Boundary Conditions

(1) Wall boundary: *u* is the velocity in the direction perpendicular to the wall (*z* direction), and the velocities in other directions were *v*, *w*. The change rate of turbulent kinetic energy and turbulent kinetic energy dissipation energy are zero in the *z* direction. The specific equations are as follows:

No slip wall:

*u* = 0, *v* = 0, *w* = 0, *k* = 0, *ε* = 0 (7)

Free slip wall

*u* = 0, (8)

(9) $$\frac{\partial v}{\partial z}=0,{}_{}\frac{\partial w}{\partial z}=0,{}_{}\frac{\partial \varepsilon }{\partial z}=0,{}_{}\frac{\partial k}{\partial z}=0,$$

(10) $$\frac{\partial v}{\partial z}=0,{}_{}\frac{\partial w}{\partial z}=0,{}_{}\frac{\partial \varepsilon }{\partial z}=0,{}_{}\frac{\partial k}{\partial z}=0,$$

In Equations (9) and (10), *z* represents the direction perpendicular to the free surface and *u* represents the velocity component in the *z* direction.

(2) Inlet boundary: each nozzle was set as an inlet boundary, and the volume fraction of water was set to 0; the mass flow rate of air was set to 1.76 Nm^3^·h^−1^. The velocity direction of air was set perpendicular to the interface, the volume fraction of air was set to 1 and the temperature was set to 25 °C.
(3) Opening boundary: the opening boundary was set to above the RH vacuum chamber. The opening option was set to training, and the pressure option was set to opening.

## 3. Results

### 3.1. Water Model Experiment

#### 3.1.1. Influence of Gas Flow on Circulating Flow Rate

**Figure 5:**
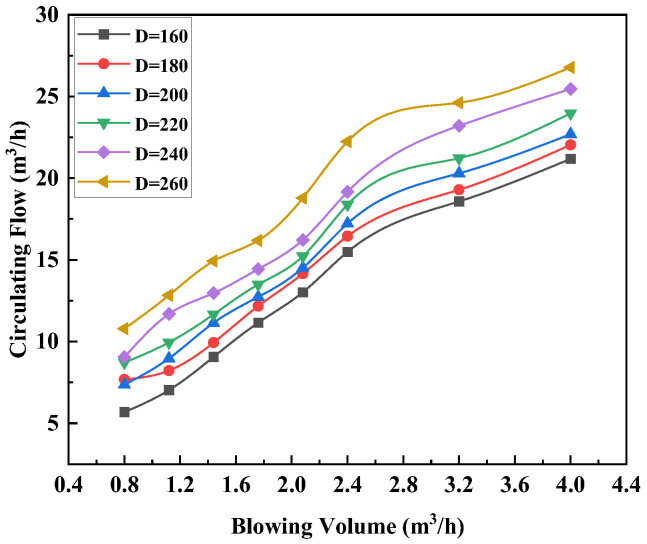
Relationship between the gas flow rate and the circulating flow rate.

It can be seen from Figure 5 that the circulating flow rate increased with the increas of the gas flow rate at different immersion depths of the immersion tube.

An inflection point appeared at Q_gas_ = 2.4 m^3^/h, indicating that Q_gas_ = 2.4 m^3^/h was the critical value of gas flow rate. When the gas flow rate reached 2.4 m^3^/h, the bubbles dissolved in molten steel and gradually reached saturation. When Q_gas_ > 2.4 m^3^/h, the influence of the gas flow rate on the circulating flow rate gradually decreased, and the increase rate of the circulating flow rate decreased as well. During the RH refining process, the driving gas blown into the upleg was the power source that drove the circulating flow of molten steel. The gas formed a bubble group in the upleg. The bubbles worked on the molten steel around it during the rising process, driving the molten steel to the vacuum chamber through the upleg, so as to achieve the purpose of vacuum refining. 

When the driving gas flow rate was small, the rising tube formed a bubble group with a small density and the gas in the molten steel did not reach saturation. With an increased gas flow rate, the density of bubbles in the upleg increased, which could effectively improve the driving ability of bubbles, thereby increasing the circulating flow rate of molten steel. When the gas flow rate reached a critical value, the bubbles in the molten steel reached saturation. It was not obvious to enhance the effect of gas flow rate on the bubble driving force.

#### 3.1.2. Effect of Nozzle Layout on Circulating Flow Rate 

In this paper, four different nozzle layouts were investigated, including the staggered layout, one-sided layout, symmetrical layout and one-row layout, as shown in Figure 3. There were 6 different layouts for the staggered layout, one-side layout and symmetrical layout, and 4 kinds of layouts for the one-row layout, for a total of 22 kinds of layout modes, which are shown in Table 4.

**Figure 6:**
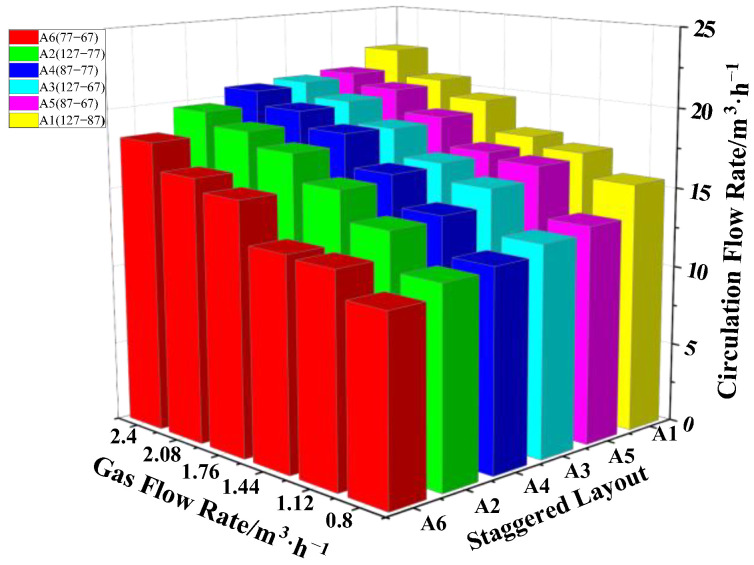
Influence of the gas flow rate on the circulating flow rate under the staggered layout.

There were 6 different layouts in the staggered layout, including A1 (127-87), A2 (127-77), A3 (127-67), A4 (87-77), A5 (87-67) and A6 (77-67), where the distances between the vacuum chamber and the nozzle (d) were d_A1_ = 426 mm, d_A2_ = 431 mm, d_A3_ = 436 mm, d_A4_ = 451 mm, d_A5_ = 456 mm and d_A6_ = 461 mm, respectively. In Figure 6, at the same gas flow rate for different staggered layouts, the variation of the circulation flow rate was QA1 > QA5 > QA4 > QA3 > QA2 > QA6.

The circulating flow rate did not increase with distance between the vacuum chamber and the nozzle. On the other hand, the circulating flow rate increased with the gas flow rate. The main factor affecting circulating flow was not the distance between the vacuum chamber and the nozzle, but the gas flow rate. The circulating flow was the maximum in the A1 (127-87) layout, Q _gas_ = 2.4, which was Q_A1_ = 22.4 m^3^/h.

**Figure 7:**
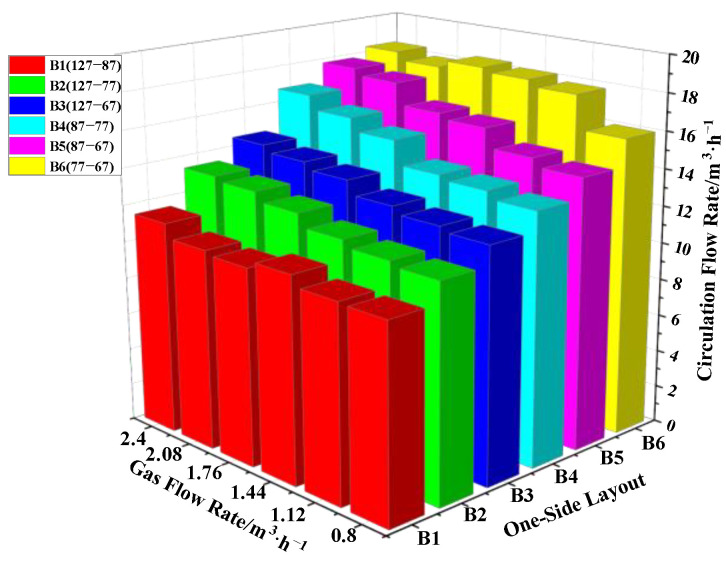
Influence of the gas flow rate on the circulating flow rate under One-Side layout.

Six different layouts were set in the one-side arrangement: B1 (127-87), B2 (127-77), B3 (127-67), B4 (87-77), B5 (87-67) and B6 (77-67) layout, where the distances between the vacuum chamber and the nozzle were d_B1_ = 426 mm, d_B2_ = 431 mm, d_B3_ = 436 mm, d_B4_ = 451 mm, d_B5_ = 456 mm and d_B6_ = 461 mm, respectively. In Figure 7, the circulating flow rate changed little with increasing gas flow rate for the same layout.

However, circulating flow rate increased obviously with increasing distance between the vacuum chamber and the nozzle. Thus, the distance between the vacuum chamber and the nozzle was the main factor to improve the circulating flow rate in the type B layout. The gas flow rate had little effect on the circulating flow rate. The maximum circulating flow rate was obtained in the B6 (77-67) layout, Q_gas_ = 2.4 m^3^/h, and its value was Q_B6_ = 18.4 m^3^/h.

**Figure 8:**
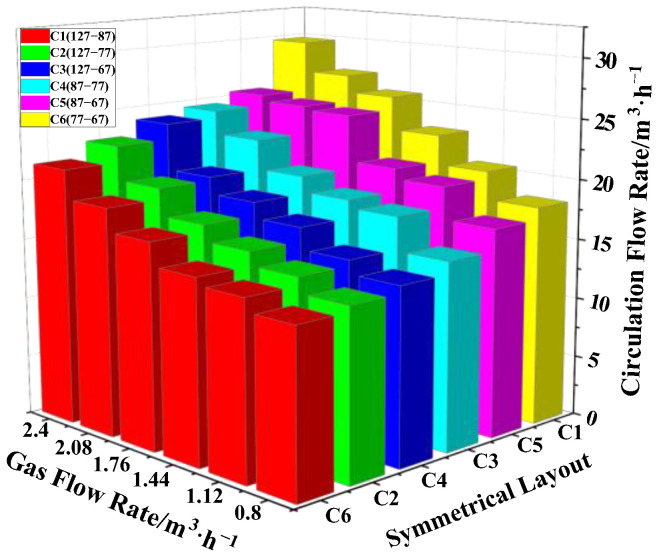
Influence of the gas flow rate on the circulating flow rate under symmetrical layout.

The symmetrical layout included 6 different arrangements. These were C1 (127-87), C2 (127-77), C3 (127-67), C4 (87-77), C5 (87-67) and C6 (77-67), where the distances between the vacuum chamber and the nozzles were d_C1_ = 426 mm, d_C2_ = 431 mm, d_C3_ = 436 mm, d_C4_ = 451 mm, d_C5_ = 456 mm and d_C6_ = 461 mm, respectively. It can be seen from Figure 8 that the variation of the circulating flow rate was Q_C1_ > Q_C5_ > Q_C3_ > Q_C4_ > Q_C2_ > Q_C6_ for different layouts with the same gas flow rate.

Circulating flow did not change linearly with distance between the vacuum chamber and the nozzle. Moreover, circulating flow rate increased with the gas flow rate for the same layout in type B. The maximum circulating flow rate reached for the C1 (127-87) layout was 29.8 m^3^/h, Q_gas_ = 2.4 m^3^/h.

**Figure 9:**
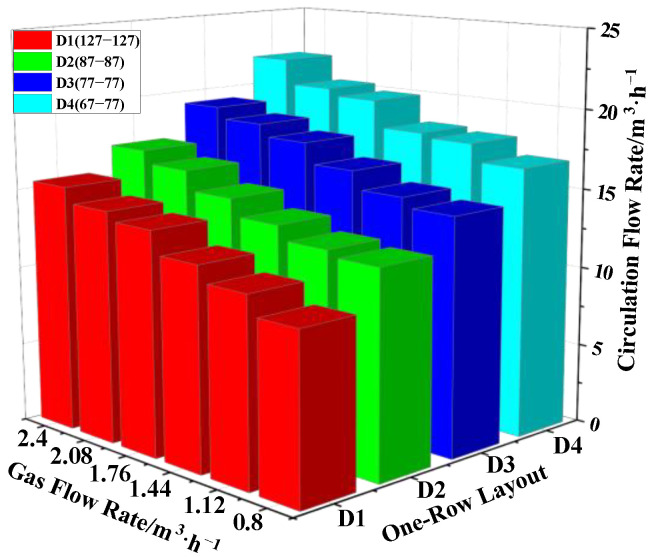
Influence of the gas flow rate on the circulating flow rate under One-Round layout.

The one-row layout had four different layouts, which were D1 (67), D2 (77), D3 (87) and D4 (127), where the distance between the vacuum chamber and the nozzle was d_D1_ = 406 mm, d_D2_ = 446 mm, d_D3_ = 456 mm and d_D4_ = 466 mm, respectively. As can be seen from Figure 9, under the same layout, the circulating flow rate increased when the gas flow rate increased. For the same gas flow rate, the variation in the circulating flow rate was: Q_D4_ > Q_D3_ > Q_D2_ > Q_D1_. The circulating flow rate increased with increasing distance between the vacuum chamber and the nozzle. Both the distance between the vacuum chamber and the nozzle and the gas flow rate have obvious promoting effects on the circulating flow rate. In contrast, the distance between the vacuum chamber and the nozzle had a greater effect on the circulating flow, as shown on Figure 9. The maximum circulating flow rate was 20.9 m^3^/h for the D1 (67-67) layout, when Q_gas_ was 2.4 m^3^/h.

**Figure 10:**
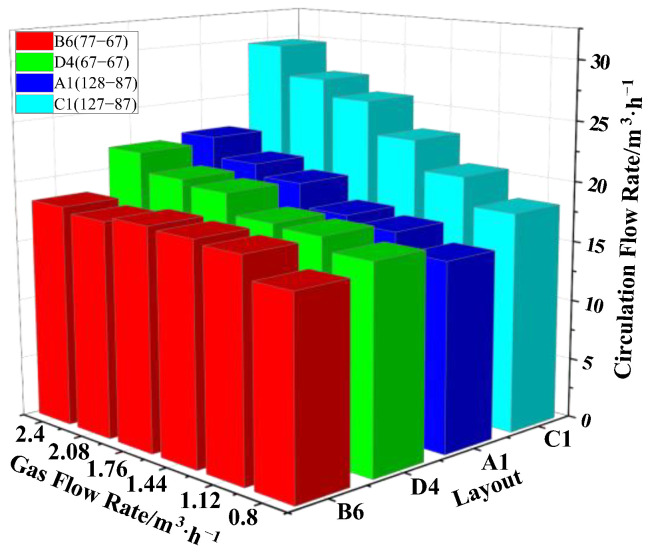
Comparison of effects of the nozzle layout on the circulating flow rate.

Out of the four types of nozzle layouts, the layouts with the largest circulating flow, in decreasing order, were A1, B6, C1 and D4. In Figure 10, these four layouts were compared. The circulating flow rate of the symmetrical layout C1 (127-87) was 29.8 m^3^/h, which was much larger than the other three, which were Q_A1_ = 22.4 m^3^/h, Q_B6_ = 18.4 m^3^/h and Q_D4_ = 20.9 m^3^/h. Obviously, the symmetrical layout C1 (127-87) was the optimal layout in this paper.

### 3.2. Numerical Simulation Verification 

In order to further explain the influence of the nozzle layout on the circulating flow rate and verify the accuracy of the water model experimental results, ANSYS simulation software was used to study the influence of the nozzle layout on the flow field distribution in the ladle.

#### 3.2.1. Effect of Gas Flow Rate on Fluid Flow in Refining Ladle 

**Figure 11:**
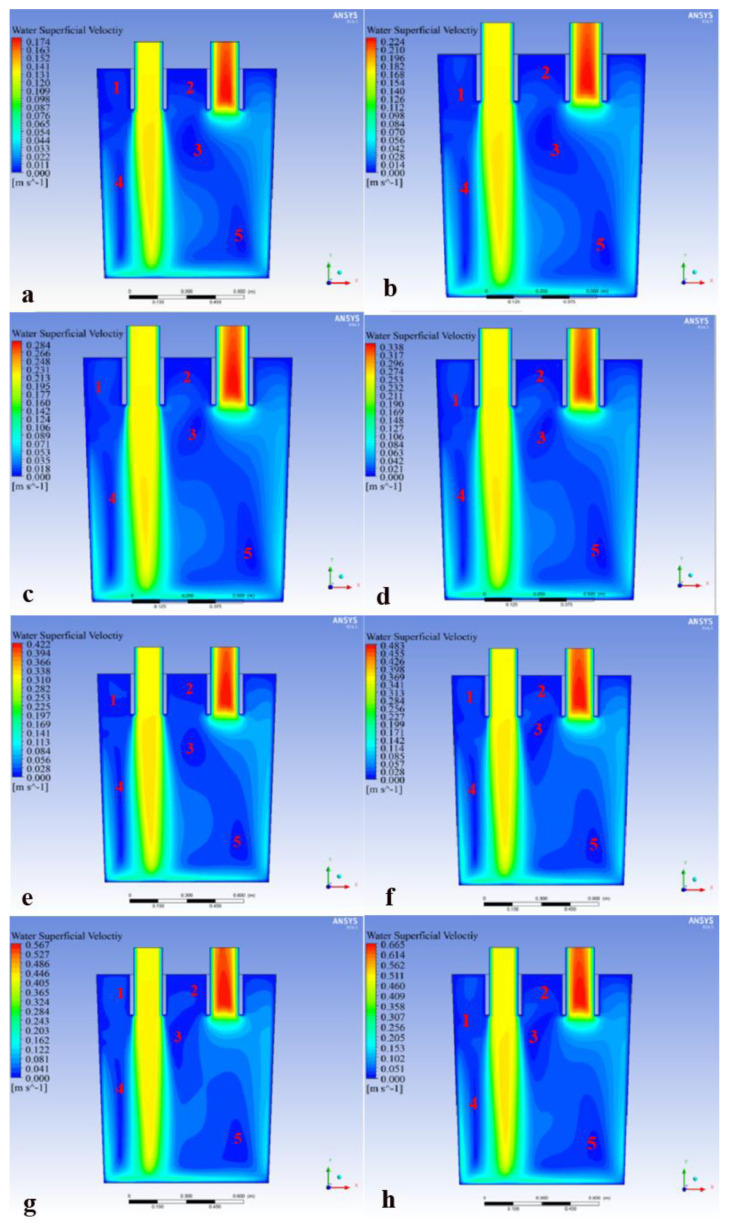
Velocity cloud diagram of the RH refining ladle central section under different gas flow rates: (**a**) Q_gas_ = 0.8 m^3^/h, (**b**) Q_gas_ = 1.2 m^3^/h, (**c**) Q_gas_ = 1.44 m^3^/h, (**d**) Q_gas_ = 1.76 m^3^/h, (**e**) Q_gas_ = 2.08 m^3^/h, (**f**) Q_gas_ = 2.4 m^3^/h, (**g**) Q_gas_ = 3.2 m^3^/h and (**h**) Q_gas_ = 4.0 m^3^/h.

Figure 11 shows the velocity cloud diagram of the central section of the RH refining furnace ladle for different gas flow rates while using the staggered layout A1 (127-87).

It can be seen that there were five blind zones in the ladle for different gas flow rates (the red numbers 1, 2, 3, 4 and 5 in Figure 11 indicate the different blind zones). The No.1 blind zone was the area between the upper left side of the downleg and the ladle wall. The No.2 blind zone appeared in the area between the downleg and the upleg. The No.3 blind zone was presented in the area directly below the middle of the downleg and the upleg. The No.4 blind zone was in the area between the lower left side of the downleg and the ladle wall. The No.5 blind zone also appeared between the lower part of the upleg and the ladle wall. Comparing the velocity cloud diagram of the ladle with the A1 (127-87) layout at different gas flow rates, it can be seen from Figure 11a–h that with the increase of the gas flow rate the No.1 blind zone first decreased and then increased; the area of the No.1 blind zone was the smallest when Q_gas_ = 3.2 m^3^/h. The change in the gas flow rate had little effect on the blind zones of No.2 and No.4. The areas of the blind zones of No.3 and No.5 decreased with the increase of the gas flow rate. When Q_gas_ = 2.4 m^3^/h, a break appeared at the low velocity zone between the No.3 and No.5 blind zones, which played a positive role in the flow of molten steel in the ladle. However, the increase or decrease of the gas flow rate could not change the distribution of the flow blind zone in the ladle. 

**Figure 12:**
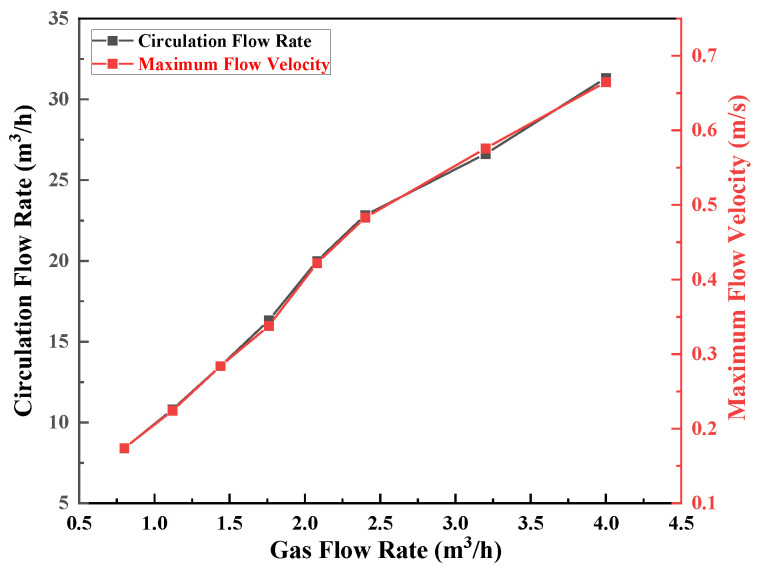
Influence of the gas flow rate on the circulating flow rate and the maximum flow rate of molten steel in the staggered layout 127-87(A1).

In Figure 12, the circulating flow rate, and the maximum flow velocity, are seen increasing with increasing gas flow rate. When the gas flow rate reached 2.4 m^3^/h, there was an inflection point, indicating that the gas flow rate in the molten steel was close to saturation. The carrying efficiency of the gas flow rate per unit volume to molten steel decreased when Q_gas_ > 2.4 m^3^/h. At this time, the increase of the circulating flow rate will gradually decrease with the increase of the gas flow rate, which coincides well with the results of the water model experiment.

#### 3.2.2. Influence of Nozzle Layout on Fluid Flow in Refining Ladle 

From the results of the water model, it can be seen that among the four nozzle layouts, the layouts with the largest circulating flow, in decreasing order, were the staggered layout 127-87(A1), one-side layout 77-67(B6), symmetrical layout 127-87(C1) and one-row layout 67(D4).

**Figure 13:**
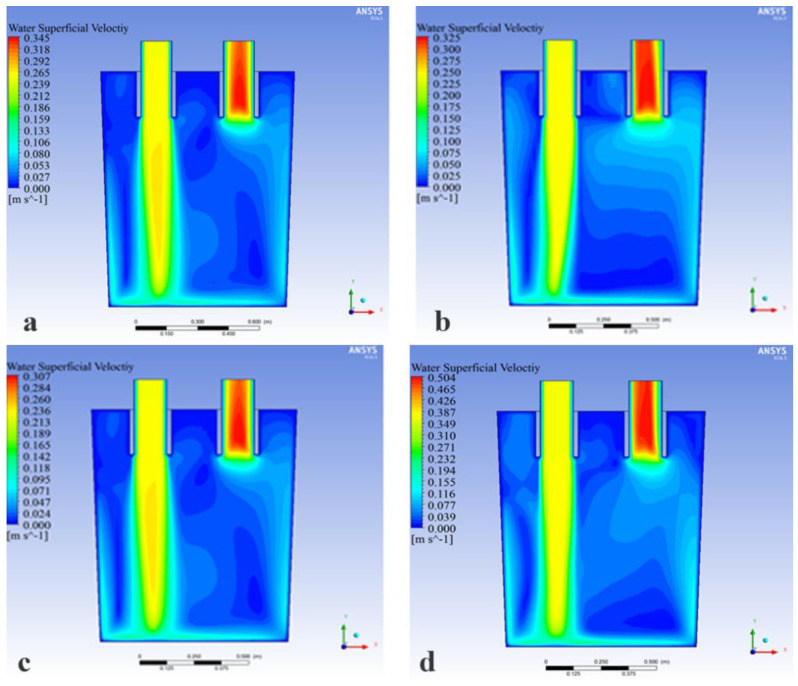
Velocity cloud diagram of the RH refining ladle center section under different nozzle layouts: (**a**) 127-87 Staggered Layout, (**b**) 77-67 One-Side Layout, (**c**) 127-87 Symmetrical Layout, (**d**) 67 One-Row Layout.

Figure 13 shows the velocity cloud diagram of the RH ladle center section from above for four layouts when the immersion tube was immersed in 220 mm and the blowing volume was 1.76 m^3^/h.

**Figure 14:**
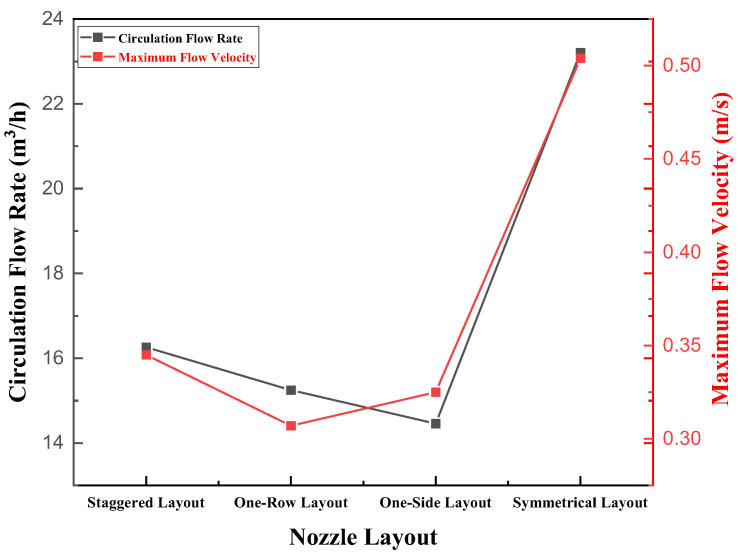
Calculated circulation flow rate of the RH refining ladle under different nozzle layouts.

It can be seen from Figure 13a,d that there were five blind zones in the ladle when the nozzle layout was in the staggered layout 127-87(A1) and the one-row layout 67(D4). The distribution and area of the blind zones for these two nozzle layouts were similar. Among them, the No.1 blind zone: the area between the upper left side of the downleg and the ladle wall, the No.2 blind zone: the area between the upleg and the downleg, the No.3 blind zone: the area directly below the middle of the upleg and the downleg, the No.4 blind zone: the area between the lower left side of the downleg and the ladle wall and the No.5 blind zone: the area below the upleg and the ladle wall. In the one-side layout 77-67 (B6), the area of the No.1 blind zone in the ladle was reduced and the No.3 blind zone disappeared, whereas the B6 layout made the area of the No.5 blind zone increase greatly, which led to a worse flow at the bottom of the ladle and was harmful to the mixing of molten steel, as shown in Figure 13b. The area of the No.5 blind zone became smaller when the nozzle layout was in the symmetrical layout 127-87(C1). Furthermore, the No.1 and No.3 blind zones in the ladle disappeared. The area of the blind zone in the ladle decreased obviously, as shown in Figure 13c. The relationship between the nozzle layouts and the circulating flow rate and the maximum flow rate is shown in Figure 14, and the values for which are: Q_Staggered_ = 16.2 m^3^/h, V_Staggered_ = 0.35 m/s, Q_One-Side_ = 15.3 m^3^/h, V_One-Side_ = 0.50 m/s, Q_One-Row_ = 14.5 m^3^/h, V_One-Row_ = 0.31 m/s, Q_Symmetrical_ = 23.2 m^3^/h and V_Symmetrical_ = 0.50 m/s, respectively.

**Figure 15:**
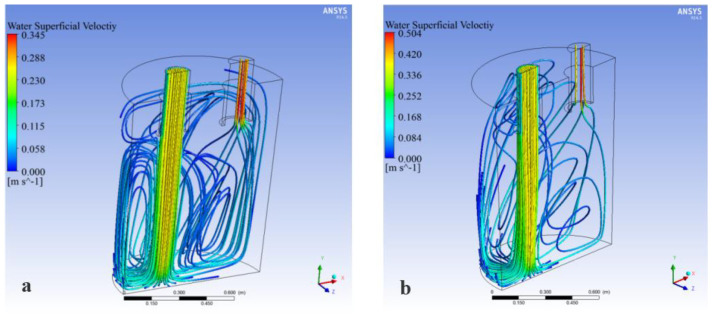
Fluid streamline in the staggered 127-87 RH refining ladle: (**a**) staggered layout 127-87, (**b**) symmetrical layout 127-87.

As can be seen from the above, regardless of the circulating flow rate or the flow field distribution, the symmetrical layout 127-87(C1) was the optimal layout. However, at present, the type A layout was common in production. In order to further reveal the influence of the staggered layout (type A) and the symmetrical layout (type C) on the fluid flow, the fluid streamline diagram of the RH refining device was analyzed, as shown in Figure 15.

It can be seen from Figure 15a that the flow of the upper fluid in the transverse direction of the ladle was mainly composed of two parts in the staggered layout 127-87 (A1). First, due to the suction effect of the upleg, the fluid near the upleg nozzle flowed into the upleg and entered the vacuum chamber. In addition, most of the remaining fluid flowed to the downstream due to the traction of the downleg. In the middle of the ladle, due to the traction of the descending stream and the obstruction of the side wall of the ladle, a circulation flow was formed on the upper and lower sides near the downleg. The flow velocity near the circulation center was slow, and there was a blind zone. At the bottom of the ladle, due to the large speed when leaving the downleg orifice, the stream flowed radially around the bottom wall of the ladle after impact. A blind zone also formed near the bottom of the ladle. Compared with the staggered layout (type A), it can be seen from Figure 15b that the velocity of the flow in the symmetrical layout 127-87(C1) was much higher than in the staggered layout 127-87(A1). The velocity direction consistency of flow in the upper part of the ladle and the lower part of the riser was worse when the nozzle was in the type C layout. There was no circulation flow near the upleg and the downleg, which indicated that the mixing effect in the ladle was better.

In summary, the nozzle layout had a great effect on the circulation flow rate and the flow field distribution of the ladle. When the nozzle layout was in the staggered 127-87 layout, the circulation flow rate and the flow velocity were both maximized. The results of the numerical simulation were consistent with the results of the water model. Therefore, it can be concluded that the nozzle layout was the key to increasing the circulating flow rate and optimizing the fluid flow in the ladle. The symmetrical layout 127-87 was the best of those analyzed.

## 4. Discussion

According to the principle of the pneumatic lifting pump, the gas pumped into the upleg through the nozzles was the power source for driving the liquid steel circle. The working stroke and carrying capacity of the bubbles were important factors affecting the circulating flow rate. The working stroke was determined by the distance (d) between the nozzle and the vacuum chamber, and the carrying capacity was related to the specific surface area and density of the bubbles.

The type B layout distributed on one side of the upleg led to an uneven distribution of bubbles. Within a short distance of the bubbles rising, they quickly collided and grew to form larger bubbles, which reduced the density and specific surface area of the bubbles, which greatly weakened the ability of each bubble to carry molten steel. It greatly limited the ability of the bubbles to carry molten steel, as the carrying capacity of the bubble was greatly reduced in the type B layout in a short time. Therefore, increasing the gas flow rate has little effect on the circulating flow rate in the type B layout. Bubble stroke was the main factor for improving the circulating flow rate. 

The nozzles of the Type D layout were evenly distributed around the upleg. Compared with the type B layout, the probability of longitudinal collision of bubbles in the type D layout was much smaller. Although the transverse spacing between the nozzles in the type D layout was short, due to the small transverse velocity of the bubbles, the probability of transverse collision of bubbles was much smaller than that of longitudinal collision. Therefore, in general, the probability of bubble collision in the D layout was less than that in the B layout. The same goes for the type B layout, where bubble stroke was the main factor for improving the circulating flow rate.

Different degrees of dislocations existed in the nozzles of the type A and the type C layouts, which meant the nozzles were far away from each other. It effectively reduced the probability of bubble collision during the process of the bubbles rising, so that the bubbles in upleg maintained a high carrying capacity of molten steel. The working stroke and carrying capacity of the bubbles to molten steel were important factors affecting the circulating flow rate. However, with the increase of bubble stroke, the probability of bubble collision and growth also increased. Under different nozzle layouts, the decisive factors of circulating flow are different. In the type A and C layouts, Q_Amax_ = Q_A1_ = 22.4 m^3^/h, Q_Amin_ = Q_A6_ = 18.2 m^3^/h, Q_Cmax_ = Q_C1_ = 29.8 m^3^/h and Q_Cmin_ = Q_C6_ = 21.3 m^3^/h, where d_A6_ = d_C6_ = 461 mm > d_A1_ = d_C1_ = 426 mm. When the circulating flow rate reached the maximum value, the distance between the vacuum chamber and the nozzle was shortest. The minimum circulating flow rate value was obtained at the longest distance between the vacuum chamber and the nozzle. Thus, it could be concluded that the bubble carrying capacity was a main factor for the circulation flow rate in the type A and C layouts.

For type A and type C, bubble carrying capacity was the main factor for the circulation flow rate, where Q_max_ is Q_A1_ = 22.4 m^3^/h and Q_C1_ = 29.8 m^3^/h. For type B and type D, bubble stroke was the main factor for the circulation flow rate, where Q_max_ was Q_B6_ = 18.4 m^3^/h, Q_D4_ = 20.9 m^3^/h. Compared to the B6, D4 and A1 layouts, the circulation flow rate of the C1 layout was 61.96%, 42.6% and 33% higher, respectively. When other conditions were the same, the maximum circulating flow of the A layout and the C layout were larger than that of the B layout and the D layout. It is concluded that for the same conditions, the carrying capacity of bubbles had a greater effect on improving the circulating flow rate than the bubble stroke.

## 5. Conclusions

1. The circulating flow rate increased as the gas flow rate increased, where the critical value of the gas flow rate was 2.4 m^3^/h.
2. Among the four types of layouts, the symmetrical layout had the most obvious advantages. Compared to the one-side layout, one-row layout and staggered layout, the circulation flow rate of the symmetrical layout was 61.96%, 42.6% and 33% higher, respectively. Out of the 22 layouts, the 127-87 symmetrical layout was the best of those analyzed, for which the circulating flow rate reached 29.8 m^3^/h and the area of the blind zone was the smallest and the mixing effect of the molten steel was the best.
3. When the nozzle layout was using the one-side layout and the one-row layout, the main factor for improving the circulating flow rate was the working stroke of the bubbles. When nozzles were using the staggered layout and the symmetrical layout, the carrying capacity of bubbles was the main factor for improving the circulating flow rate.
4. The working stroke and carrying capacity of bubbles were important factors affecting the circulating flow rate. For the same conditions, the carrying capacity of bubbles had a greater effect on improving the circulating flow rate than the bubble working stroke.

## Figures and Tables

**Table 1:** Main parameters for the model and the prototype.

Item	Height of Ladle/mm	Bottom Diameter of Ladle/mm	Top Diameter of Ladle/mm	Length of Snorkels/mm	Internal Diameter of Snorkels/mm	Diameter of Vacuum Vessel /mm
Prototype	3423	2571	2808	1599	450	1650
Water Model	1141	857	936	533	150	550

**Table 2:** Physical parameters of the water model and the prototype.

Facility	Item	Density/(kg·m^−3^)	Temperature/K
Water model Water	Water	1000	298
	Air	1.292	
Prototype	Liquid steel	7000	1873
	Argon	1.782	

**Table 3:** Experimental program.

Circulation Flow Rate /m^3^ h^−1^	Immersion Depth/mm	Nozzle Layout
0.8/1.12/1.44/1.76/2.08/2.4	220	Staggered Layout/One-side Layout/ Symmetrical Layout/One-row Layout

**Table 4:** Layout of nozzles.

Layout	Staggered Layout					
Nozzle layout	127-87(A1)	127-77 (A2)	127-67 (A3)	87-7 (A4)	87-67 (A5)	77-67 (A6)
**Layout**	**Symmetrical Layout**					
Nozzle layout	127-87(C1)	127-77 (C2)	127-67(C3)	87-77 (C4)	87-67 (C5)	77-67(C6)
**Layout**	**One-Side Layout**					
Nozzle layout	127-87 (B1)	127-77(B2)	127-67(B3)	87-77 (B4)	87-67 (B5)	77-67 (B6)
**Layout**	**One-Row Layout**					
Nozzle layout	127-127 (D1)		87-87 (D2)	77-77 (D3)	67-67 (D4)	

## Data Availability

Data sharing not applicable.
